# Epistaxis Rates and Health Care Utilization in Patients With a Ventricular Assist Device

**DOI:** 10.1002/oto2.132

**Published:** 2024-04-12

**Authors:** Eric Rohe, Sarah Schmoker, Kaeli Samson, Kristy Carlson, Jayme Dowdall

**Affiliations:** ^1^ Department of Otolaryngology Head and Neck Surgery, College of Medicine University of Nebraska Medical Center Nebraska Nebraska USA; ^2^ Department of Biostatistics, College of Public Health University of Nebraska Medical Center Omaha Nebraska USA

**Keywords:** epistaxis, ventricular assist device

## Abstract

**Objective:**

Identify baseline epistaxis rates and epistaxis‐related health care utilization trends in the ventricular assist device (VAD) population.

**Methods:**

Single center, retrospective cohort study consisting of chart review of adult VAD patients. Analysis of descriptive statistics was assessed using *χ* ^2^ tests, independent sample *t* tests, or Fisher's exact when expected counts were low. Logistic regression was used to assess associations between epistaxis and variables of interest.

**Results:**

Two hundred ninety patients were included in the analysis. Ninety‐eight (33.8%) patients developed epistaxis and 84 (29.0%) received medical attention. Patients with gastrointestinal (GI) bleeding had increased rates of epistaxis (42.4% vs 29.0%). Logistic regression analysis found GI bleeding to have an adjusted odds of developing epistaxis of 1.94 (95% confidence interval [CI]: 1.12‐3.37) and kidney disease to have an adjusted odds of 1.83 (95% CI: 1.06, 3.13).

**Discussion:**

VAD implantation improves survival and quality of life but also carries significant bleeding risks. At our institution, 29% of VAD patients received medical attention for epistaxis. GI bleeding and kidney disease were found to have increased adjusted odds of developing epistaxis. Fifty‐nine percent of epistaxis events occurred while inpatient and 32.8% of events were seen in the emergency department.

**Implications for Practice:**

VAD patients are an at‐risk group that could potentially benefit from preventative nasal hydration regimen.

Ventricular assist device (VAD) implantation in patients with end‐stage heart failure has been shown to improve both survival and quality of life^1^, ^2^ and can be used as either a bridge to transplant or for permanent use (destination therapy). Implantation of a VAD carries risk of both thrombotic and bleeding complications. Biomaterials used in the lining of a VAD come into contact with blood and cause a hypercoagulable state. These circumstances increase the propensity for pump thrombosis and complications, including embolic stroke, device failure, and reduced survival.^1^, ^3^

Combination anticoagulation and antiplatelet therapy are typically used to mitigate the hypercoagulability risks, but these medications are not without risk themselves. Hemorrhagic complications are frequently seen as a complication of VAD therapy. Bleeding in the VAD population most often occurs at mucocutaneous sites, with epistaxis being the second most commonly reported location.^4^ A 2018 study found bleeding was responsible for 29.4% of emergency department visits in the VAD population, 31.3% of which were epistaxis‐related.^4^ Anticoagulation medications alone increase the bleeding risk as 10% to 17% of patients on long‐term vitamin K antagonist therapy have been shown to develop epistaxis.^5^ Flow dynamics of the VAD, development of arteriovenous malformations and acquired von Willebrand disease add to the increased risk of bleeding.^1^, ^3^, ^5^ Epistaxis can range from minor, self‐limiting episodes to life‐threatening bleeding requiring hospitalization, blood transfusions, and/or operative management. VAD patients are at higher risk of recurrent epistaxis episodes and major complications due the previously discussed reasons. To our knowledge, no studies to date have sought to evaluate the rate of epistaxis in these patients or the impact in terms of health care utilization associated with epistaxis in the VAD patient population.

## Methods

This single‐institution, retrospective chart review was approved by our institution's institutional review board (IRB) (#0327‐20‐EP). The requirement for informed consent was waived by the IRB. The criteria for inclusion was age 19 years or older and patient undergoing VAD placement from January 1, 2014, through March 31, 2020, at our institution. A list of patients meeting this criteria was generated by our institution's cardiothoracic team. Chart review of each patient was performed through our institution's Epic system. Data recorded included demographic data, device data, medical and surgical history, clinical encounter data, medications, and lab data. Study data were collected and managed using Research Electronic Data Capture tools hosted at our institution.

Initial patient‐level data was collected at the time of VAD implantation. All patients undergoing VAD placement had a diagnosis of heart failure. Additional data collected included patient demographics, type of VAD, the goal of VAD therapy, co‐morbidities, prior heart surgeries, and prior pacemaker or implantable cardioverter‐defibrillator placement and this is found in Table 1. During chart review, it was also noted whether or not patient developed any issues with gastrointestinal (GI) bleeding. Further review was aimed at identifying epistaxis events and details surrounding these events. If patient underwent VAD explantation or heart transplant, no further chart review was performed after this date. For the general patient‐level analysis, patients were classified as having at least 1 epistaxis event if they had at least 1 home or hospital event. They were otherwise classified as having no epistaxis events if they did not have a home or hospital epistaxis event. The dichotomization of epistaxis events in the patient‐level analysis allowed for events to be summarized at a patient‐level to avoid statistical dependence. All patient data were captured at the time of VAD implant with the exception of developing GI bleeding.

**Table 1:** Patient Characteristics

Column1	+ Epistaxis n (%)	No epistaxis n (%)	*P* value
Age (mean ± SD)	55.7 ± 15.0	58.1 ± 12.0	.16^a^
Sex			.18
Male	78 (35.9)	139 (64.1)	
Female	20 (27.4)	53 (72.6)	
VAD type			.76^b^
HeartMate II	53 (36.6)	92 (63.5)	
HeartMate 3	35 (31.0)	78 (69.0)	
HeartWare	9 (33.3)	18 (66.7)	
Total artificial heart	1 (20.0)	4 (80.0)	
Goal of VAD therapy			.68^b^
Destination therapy	63 (34.8)	118 (65.2)	
Bridge to transplant	32 (33.0)	65 (67.0)	
Unknown	3 (50.0)	3 (50.0)	
Co‐morbidities			
Hypertension			.64
Yes	70 (34.7)	132 (65.4)	
No	28 (31.8)	60 (68.2)	
Hyperlipidemia			.59
Yes	56 (32.6)	116 (67.4)	
No	42 (35.6)	76 (64.4)	
Cardiomyopathy			.64
Yes	89 (34.2)	171 (65.8)	
No	9 (30.0)	21 (70.0)	
Coronary artery disease			.70
Yes	62 (34.6)	117 (65.4)	
No	36 (32.4)	75 (67.6)	
Atrial fibrillation			.42
Yes	41 (36.6)	71 (63.4)	
No	57 (32.0)	121 (68.0)	
COPD			.25
Yes	28 (39.4)	43 (60.6)	
No	70 (32.0)	149 (68.0)	
Prior CVA			.52
Yes	11 (39.3)	17 (60.7)	
No	87 (33.2)	175 (66.8)	
Diabetes mellitus			.52
Yes	38 (36.2)	67 (63.8)	
No	60 (32.4)	125 (67.6)	
Peripheral vascular disease			.92
Yes	8 (34.8)	15 (65.2)	
No	90 (33.7)	177 (66.3)	
Kidney disease			.06
Yes	49 (39.8)	74 (60.16)	
No	49 (29.3)	118 (70.7)	
Prior malignancy			.18
Yes	11 (25.0)	33 (75.0)	
No	87 (35.4)	159 (64.6)	
Tobacco use			.34
Yes	35 (37.6)	58 (62.4)	
No	63 (32.0)	134 (68.0)	
Current smoker (other)			.13
Yes	9 (50.0)	9 (50.0)	
No	89 (32.7)	183 (67.3)	
Current or prior history of alcoholism			.39
Yes	5 (25.0)	15 (75.0)	
No	93 (34.4)	177 (65.6)	
Depression			.26
Yes	15 (27.3)	40 (72.7)	
No	83 (35.3)	152 (64.7)	
Gastroesophageal reflux disease			.26
Yes	36 (38.3)	58 (61.7)	
No	62 (31.6)	134 (68.4)	
OSA			.34
Yes	35 (37.6)	58 (62.4)	
No	63 (32.0)	134 (68.0)	
Hypothyroidism			.25
Yes	10 (25.6)	29 (74.4)	
No	88 (35.1)	163 (64.9)	
History of DVT or pulmonary embolus			.91
Yes	7 (35.0)	13 (65.0)	
No	91 (33.7)	179 (66.3)	
Pulmonary hypertension			.22
Yes	16 (27.1)	43 (72.9)	
No	82 (35.5)	149 (64.5)	
Prior heart surgery			.54
Yes	82 (34.5)	156 (65.6)	
No	15 (30.0)	35 (70.0)	
Prior coronary artery bypass graft			.92
Yes	25 (33.3)	50 (66.7)	
No	73 (34.0)	142 (66.1)	
Pacemaker or ICD			.25
Yes	64 (31.7)	138 (68.3)	
No	34 (38.6)	54 (61.4)	
Gastrointestinal bleeding			.03^c^
Yes	39 (42.4)	53 (57.6)	
No	56 (29.0)	137 (71.0)	

*P* values from *χ* ^2^ tests unless otherwise indicated.

Abbreviations: COPD, chronic obstructive pulmonary disease; CVA, cerebrovascular accident; DVT, deep vein thrombosis; ICD, implantable cardioverter‐defibrillator; OSA, obstructive sleep apnea; SD, standard deviation; VAD, ventricular assist device.

^a^ Independent samples *t* test.

^b^ Fisher's exact test.

^c^ Statistically significant.

There were 2 types of epistaxis events in this study. The first were events that occurred in the home and were later reported during a clinic or hospital visit. These were termed home epistaxis events and data from these events was extremely limited. The second occurred at home and the patient sought medical care in a clinic or emergency department setting or occurred while the patient was in the hospital and subsequently received medical attention for this issue. Hospital events have more detailed data about the event and data collected included the location of the encounter, whether ear nose and throat (ENT) was consulted, antiplatelet, and anticoagulant medications taken in the days leading up to the event, lab values, treatments utilized, number of intervention episodes required to control epistaxis and reasons for admission if applicable. A treatment episode was described as 1 face‐to‐face encounter with a provider and included all treatment(s) attempted to control epistaxis at that time. If patients required additional face‐to‐face encounters for treatment within 7 days of each other, this was considered an additional intervention episode for the same epistaxis event. If a patient was seen by provider(s) multiple times for epistaxis but the time between evaluations was 8 days or greater, it was considered a separate epistaxis event.

For the event‐level summary data, counts and percentages of events are given for variables of interest, and are also presented with the number of patients who contributed to the event count. For the patient‐level analysis, descriptive statistics for continuous data are given as mean ± standard deviation (SD) or counts and frequencies separately for patients who did or did not have at least 1 epistaxis event. Associations between categorical variables of interest and epistaxis were assessed using *χ* ^2^ tests or Fisher's exact tests when expected cell counts were low. An independent sample *t* test was used to assess the difference in age at VAD implantation between patients who did or did not have at least 1 epistaxis event. Logistic regression was used to assess associations between epistaxis and variables of interest; variables were included in the model if they had an univariable *P* value less than .20. Adjusted odds ratios are presented with 95% confidence intervals (CIs). All analyses were performed using SAS software version 9.4 (SAS Institute Inc).

## Results

### Patient‐Level Analysis

**Figure 1:**
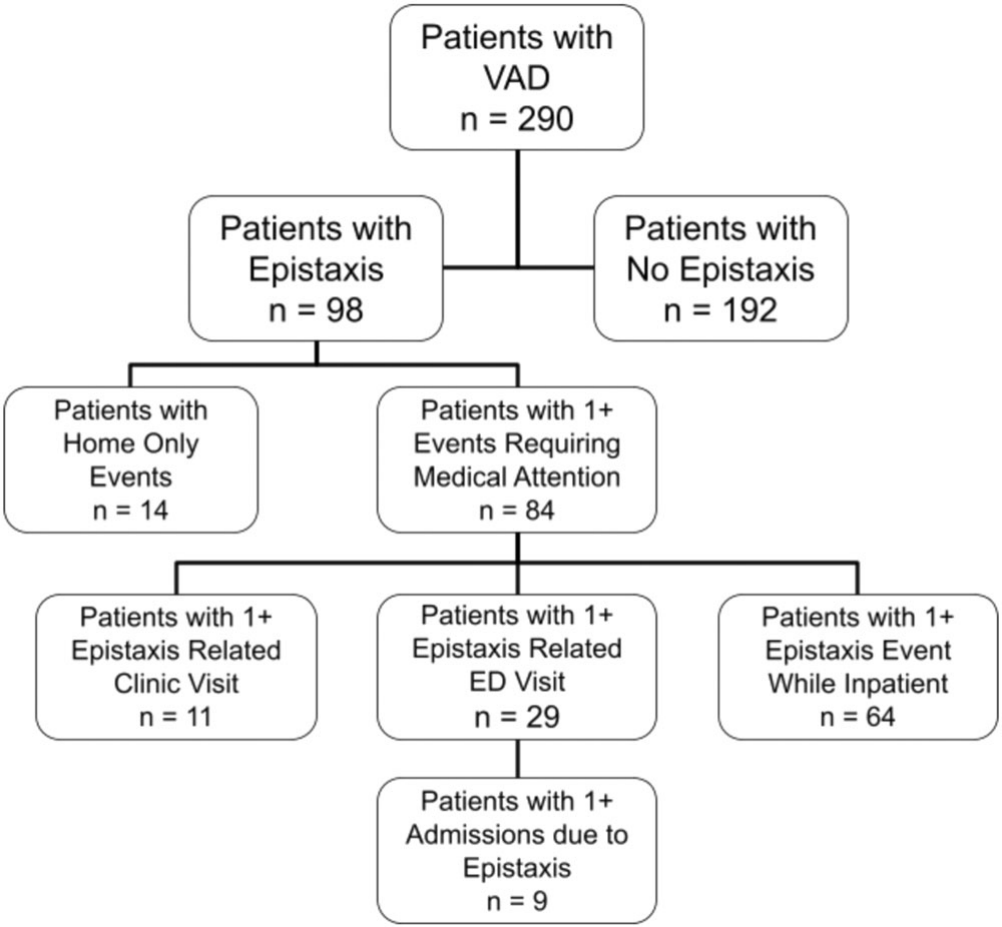
Flowsheet displaying number of patients with epistaxis events and location(s) where patients experienced 1+ treatment encounters. VAD, ventricular assist device.

A list of 296 unique patients were initially identified by our cardiothoracic team to undergo chart review. Six of these were excluded due to VAD implantation outside inclusion date range (4) and initial VAD implanted at outside institution (2). A total of 290 patients were included in the analysis and Table 1 details patient characteristics of recorded data. Descriptive statistics for continuous data were used to compare patients who had at least 1 epistaxis event and patients who did not have an epistaxis event. Of the 290 patients included in the analysis, 98 (33.8%) were found to have an epistaxis event (Figure 1). Of the 290 patients, 84 (29.0%) had an epistaxis event for which they received medical attention. There was no statistical difference in age at VAD implantation between patients who had at least 1 epistaxis event and those who did not (55.7 ± 15.0 vs 58.1 ± 12.0, *P* = .16). The proportion of patients who had at least 1 epistaxis event was not significantly different across VAD types (*P* = .76). There was a higher percentage of patients with epistaxis among patients with GI bleeding issues compared to patients who did not have GI bleeding issues (42.4% vs 29.0%, *P* = .03). No other variables were found to be associated with epistaxis.

**Figure 2:**
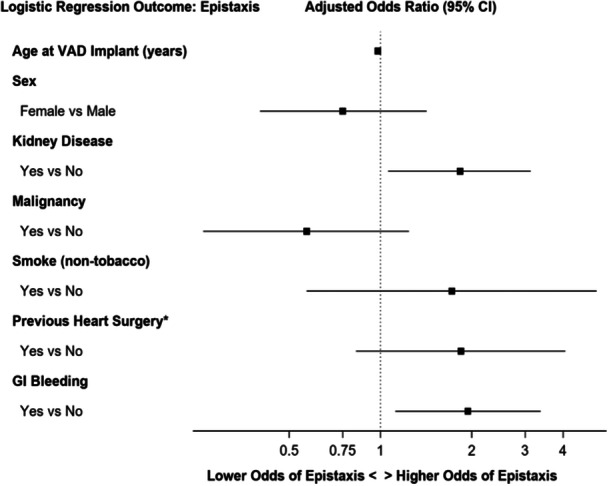
Forest plot of logistic regression. After adjusting for other variables in the model, patients with kidney disease had 1.83 (95% confidence interval [CI]: 1.06, 3.13; *P* = .03) times the adjusted odds, and patients with GI bleeding had 1.94 (95% CI: 1.12, 3.37; *P* = .02) times the adjusted odds of having at least 1 epistaxis event compared to patients without these conditions. *Excludes previous CABG/pacemaker/ICD. CABG, coronary artery bypass graft; GI, gastrointestinal; ICD, implantable cardioverter‐defibrillator; VAD, ventricular assist device.

Logistic regression was used to assess associations between epistaxis and variables of interest (Figure 2). Variables were included in the model if they had a univariable *P* value less than .20. After adjusting for the other variables in the model, patients with GI bleeding had 1.94 times the adjusted odds of having at least 1 epistaxis event than patients without GI bleeding (95% CI: 1.12‐3.37, *P* = .02). Patients with kidney disease had 1.83 times the adjusted odds of having at least 1 epistaxis event than patients without kidney disease (95% CI: 1.06‐3.13, *P* = .03). No other variables were found to be associated with epistaxis on logistic regression modeling.

### Event‐Level Analysis

Data from each epistaxis event was also gathered and analyzed. To generate descriptive statistics for events, only events that occurred within the hospital, emergency department, or clinic setting were used as this allowed for better comparison of clinical variables. Fourteen patients were excluded as they had exclusively reported home‐only events for which they did not receive medical attention. Additionally, a single patient with over 20 epistaxis‐related events was excluded from event‐level descriptive statistics due to concerns that the presence of these events could heavily skew estimates. Detailed information for this particular patient can be found in a previous case report.^6^ With this patient excluded, there were 134 epistaxis events from 83 patients. Findings are detailed in Table 2. As some patients contributed to a multiple of the 134 epistaxis events, the number of patients contributing to event counts will also be given in the following paragraphs.

**Table 2:** Event Data

Column1	n (%)
Total epistaxis events^a^	134 (100)
ENT consulted	
Yes	88 (65.7)
No	46 (34.3)
Location of event/encounter	
Clinic—Cardiothoracic	1 (0.8)
Clinic—ENT	10 (7.5)
Emergency department	44 (32.8)
Previously admitted—ICU	25 (18.7)
Previously admitted—PCU	5 (3.7)
Previously admitted—Floor	49 (36.6)
Intervention episodes required	
None	37 (27.6)
1	68 (50.8)
2	20 (14.9)
3 or more	9 (6.7)
Antiplatelet/anticoagulant medications	
Aspirin 325 mg	5 (4.1)
Aspirin 81 mg	96 (78.1)
Dipyridamole	37 (29.4)
Other antiplatelet	10 (7.9)
Warfarin	126 (94.0)
Other anticoagulant	4 (3.0)
INR (mean ± SD; n = 115)	2.7 ± 1.2
Treatments utilized	
Pressure	74 (55.2)
Topical vasoconstrictors	76 (56.7)
Nasal ointment	28 (20.9)
Nasal cautery	31 (23.1)
Nasal packing	69 (51.5)
1 Packing event	42
2+ Packing events	27
Topical thrombin matrix	11 (8.2)
Topical tranexamic acid	8 (6.0)
Operating room	3 (2.2)

One hundred thirty‐four events from 83 unique patients.

Abbreviation: ENT, ear nose and throat; ICU, intensive care unit; INR, international normalized ratio; PCU, progressive care unit.

^a^ Excludes home events and single patient with 20+ events.

ENT was consulted for 88 (65.7%) events. Sixty‐three (75.9%) of the 83 patients who experienced epistaxis events required at least 1 ENT consultation. Seventy‐nine events (59.0%) occurred while patients were already inpatient; 25 events in the intensive care unit from 24 patients, 5 events in the progressive care unit from 5 patients, and 49 events on the floor from 40 patients. Forty‐four events (32.8%) occurred in the emergency department (28 patients) and 11 events (8.2%) occurred in the clinic setting. One patient was seen in the cardiothoracic clinic for a single epistaxis event. Nine patients were seen in ENT clinic for a total of 10 epistaxis events.

Anticoagulant and antiplatelet medications taken at the time of each event along with international normalized ratio (INR) lab value, if available, were collected and analyzed (Table 2). INR data was available for 115 events. Mean INR was found to be 2.7 with an SD of 1.2. INR was found to be ≤2.5 in 60 events (44.8%) and supratherapeutic, INR >2.5, in 55 events (41.0%). When comparing patients with INR ≤2.5 at initial epistaxis event (n = 44) versus supratherapeutic INR at initial epistaxis event (n = 32), there was no difference between rates of second epistaxis event (34.1% vs 34.4%, *P* = .64). When comparing patients with INR >3.5 at initial epistaxis event (n = 13) versus patients with INR ≤3.5 at initial epistaxis event (n = 63), there was no significant difference between rates of multiple epistaxis events (30.1% vs 34.9%, *P* = 1.00). No intervention was required after evaluation in 37 (27.6%) of events. An single intervention episode, which could include multiple modalities of epistaxis treatment, was needed in 68 (50.8%) epistaxis events (45 patients). Two intervention episodes were required for 20 (14.9%) epistaxis events (17 patients), while 9 (6.7%) epistaxis events (9 patients) required 3 or more intervention episodes.

Treatments utilized and frequency of utilization were collected and analyzed (Table 2). Nasal packing was performed in 69 (51.5%) epistaxis events (46 patients). Of these 69 events, 42 needed 1 nasal packing episode (29 patients) but 27 required 2 or more nasal packing episodes (25 patients). In total, 46 (55.4%) of the 83 patients underwent at least 1 nasal packing episode. Operative control of epistaxis was required in 3 (2.2%) epistaxis events, which came from 3 separate patients.

## Discussion

Patients with a VAD require anticoagulant and antiplatelet therapy to avoid the complication of pump thrombosis, which can lead to devastating embolic stroke or device failure.^1^ These medications alone, though necessary, place patients at risk of bleeding complications. There are slight differences in manufacturer‐recommended antiplatelet and anticoagulation therapy based on the implanted device, such as the use of warfarin with goal INR of 2.0 to 2.5 in addition to aspirin for Heartmate II and 3 devices versus the use of >81 mg aspirin in addition to dipyridamole or clopidogrel for Heartware devices.^7^ The patients in our study had several types of VAD devices which could attribute in part to the differences in anticoagulation and antiplatelet regimens seen at the time of epistaxis event. Patients on long‐term vitamin K antagonist therapy alone has shown a 10% to 17% incidence of epistaxis.^5^ Device‐related factors such as acquired von Willebrand disease, thought to be due to shearing of von Willebrand factor, and development of mucosal arteriovenous malformations add to the risk of mucosal bleeding and 1 can see how this bleeding risk could potentially be even higher.^1^, ^3^ Our study further supports this theory as 33.8% of patients had at least 1 epistaxis event and 29.0% received medical attention for at least 1 epistaxis event.

The majority, 80% to 90%, of epistaxis events in the general population are spontaneous without an identifiable cause. Brown et al found a similar 75.5% rate of spontaneous epistaxis in their review of inpatient epistaxis consults in the VAD population.^5^ Brown also found 83.6% of epistaxis episodes originated on the anterior nasal septum, consistent with multiple other studies showing approximately 90% of epistaxis events coming from Kiesselbach's plexus in the general population.^1^, ^5^, ^8^ A prior study found 32.7% of inpatient epistaxis consults for VAD patients with spontaneous epistaxis required multiple interventions, which is somewhat higher than the 21.6% of epistaxis events in our study population that required more than 1 treatment episode.^5^

Hemorrhage has been found as the most common reason for presentation to the emergency department among the VAD population.^4^ Epistaxis is the second most common site of bleeding, behind GI bleeding, and has been found as the cause of 31% of bleeding‐related emergency department presentations in this population.^4^, ^9^ Our results showed that patients with GI bleeding had statistically higher rates of developing epistaxis (42.4% vs 29.0%). Logistic regression found patients with GI bleeding had 1.94 times the adjusted odds of having at least 1 epistaxis event than patients without GI bleeding. This intuitively makes sense as both the GI tract and nasal cavity are mucosal‐lined surfaces. Additionally, device‐related acquired von Willebrand disease, known to be associated with increased risk of mucosal bleeding, and development of mucosal arteriovenous malformations would thus increase the risk of bleeding at both locations.^3^, ^10^ Patients with kidney disease were also found to have 1.83 times the adjusted odds of developing epistaxis. Knowledge of these associations could assist clinicians in risk stratifying these patients.

Mean INR at the time of epistaxis events was found to be 2.7 ± 1.2 in our study. There is a well‐documented association between periods of supratherapeutic anticoagulation and increased risk of bleeding events.^11^ A prior study in 2017 by Halder et al reviewed all bleeding events in their VAD population and found a mean INR of 2.7 ± 1.2 before a bleeding event, which is identical to our findings.^11^ Management of warfarin is beyond the scope of this study but prior studies of epistaxis in non‐VAD patients have concluded it is safe to continue anticoagulation therapy from an epistaxis management perspective, provided their INR is within therapeutic or subtherapeutic range.^12^

When considering our results, this study was performed at our regional tertiary care center and many of our patients live multiple hours away from our facilities. Nosebleeds are usually nontraumatic and spontaneous, which means that many patients rush to the nearest emergency department at the beginning of significant hemorrhage from the nose. As many patients live far away, it is unlikely they would present to our institution's emergency department and may not have been captured in our study unless they happened to mention this event to staff during a future encounter. Our results are therefore likely to underestimate the total number of clinic and emergency department encounters related to epistaxis.

Finally, the majority of epistaxis events (59.0%) in our study were found to be in patients who were already inpatient for another reason. This percentage could be artificially high due to our institution being a tertiary care center with many patients living multiple hour drive distance away. Nevertheless, this number brings up a significant factor from a patient perspective. VAD patients are in general very sick with multiple co‐morbidities. They have often undergone many prior invasive and painful procedures. Epistaxis can be severe but also an extreme nuisance as patients are typically dealing with other life‐threatening conditions. Awake interventions are most commonly used to treat epistaxis in general, but this is especially true of VAD patients due to the desire to avoid anesthesia‐associated cardiac risks. Nasal cautery and nasal packing are 2 treatments frequently used to treat epistaxis, but they can also be very uncomfortable and sometimes painful for patients. Brown et al found that silver nitrate cauterization alone was associated with need for repeat interventions in the VAD population and recommended hemostatic material be considered as initial management for epistaxis in VAD patients.^5^ Nasal packing was performed in 51.5% of epistaxis events in our study, with 39.1% of these patients requiring at least a second nasal packing treatment later on. These numbers highlight epistaxis as a complication, that if decreased or prevented, could significantly improve the patient experience and decrease the necessity of uncomfortable or painful procedures. Studies have shown that simple nasal hydration regimen with nasal saline gel can cause cessation of chronic epistaxis in 93% of patients in the general population.^13^ As a nasal hydration regimen is cheap, easy to administer, and painless for the patient, this would be an ideal preventative intervention that could lead to improvement in patient outcomes and experience.

The major limitation to this study is its retrospective nature with associated inherent biases such as recall bias. Additionally, much of the data obtained was identified through chart review which relies on accurate documentation at the time of the intervention.

## Conclusion

VAD implantation carries a significant risk of both thrombotic and bleeding complications due to device‐related factors along with anticoagulant and antiplatelet medications. Epistaxis is a common bleeding complication in the VAD population and 29.0% of patients in our study received medical attention for at least 1 epistaxis event. The majority (59.0%) of events where a patient received medical attention occurred while patients were already admitted for a reason other than epistaxis. GI bleeding and kidney disease were found to be associated with increased adjusted odds of developing a nosebleed event. Epistaxis in VAD patients can be difficult to treat due to anticoagulated status along with other patient comorbidities. Nasal packing, which can be painful and traumatic to patients, was required in 51.5% of bleeding events, with 39.1% of these cases requiring at least a second packing event. Studies of epistaxis in the general population have demonstrated impressive chronic epistaxis resolution rates with a simple nasal moisturization routine. With all this information, VAD patients are an at‐risk group that could potentially benefit from a preventative nasal hydration regimen. Further studies would be needed to evaluate this potential benefit. Additionally, providing VAD patients with education and handouts regarding epistaxis management and containing ENT clinic numbers could improve patient experience and allow for enhanced epistaxis care.

## Author Contributions

**Eric Rohe**, project design, chart review, statistical analysis, manuscript writing and editing; **Sarah Schmoker**, chart review, manuscript editing; **Kaeli Samson**, Statistical analysis, manuscript writing, and editing; **Kristy Carlson**, project design, manuscript editing; **Jayme Dowdall**, project design, manuscript editing.

## Disclosures

### Competing interests

The authors declare that there is no conflict of interest.

### Funding source

None.
